# Supplementary material for: From sequence to enzyme mechanism using multi-label machine learning
Source: BMC Bioinformatics. 2014 May 19;15:150. doi: 10.1186/1471-2105-15-150 (PMC4229970; doi:10.1186/1471-2105-15-150)
Supplement: Additional file 2 — Java code of ml2db. Additional file ml2db_code.tar.gz contains the Java source code to run the multi-label machine learning experiments and save the results to database. The code’s Javadoc is included. [file 1471-2105-15-150-S2.zip › additional file 2/ml2db/ml2db/doc/mulan/classifier/transformation/package-use.html]

Uses of Package mulan.classifier.transformation


JavaScript is disabled on your browser.


- Overview
- Package
- Class
- Use
- Tree
- Deprecated
- Index
- Help

- Prev
- Next

- Frames
- No Frames

- All Classes

# Uses of Package mulan.classifier.transformation

- Packages that use mulan.classifier.transformation

  | Package | Description |
  | --- | --- |
  |  |  |
  | --- | --- |
  | mulan.classifier.transformation |  |
- Classes in mulan.classifier.transformation used by mulan.classifier.transformation

  Class and Description

  IncludeLabelsClassifier

  A multilabel classifier based on the include labels transformation.

  LabelPowerset

  Class that implements a label powerset classifier

  LabelsetPruning

  Common functionality class for the PPT and PS algorithms

  PPT.Strategy

  strategies for processing infrequent labelsets

  PrunedSets.Strategy

  strategies for processing infrequent labelsets

  TransformationBasedMultiLabelLearner

- Overview
- Package
- Class
- Use
- Tree
- Deprecated
- Index
- Help

- Prev
- Next

- Frames
- No Frames

- All Classes
